# Comment on Hardacker, C.T.; Baccellieri, A.; Mueller, E.R.; Brubaker, L.; Hutchins, G.; Zhang, J.L.Y.; Hebert-Beirne, J. Bladder Health Experiences, Perceptions and Knowledge of Sexual and Gender Minorities. *Int. J. Environ. Res. Public Health* 2019, *16*, 3170

**DOI:** 10.3390/ijerph17072310

**Published:** 2020-03-30

**Authors:** Joshua S. Jue, Mahmoud Alameddine

**Affiliations:** 1 Department of Urology, Lenox Hill Hospital, Northwell Health, Zucker School of Medicine at Hofstra/Northwell, New York, NY 11549, USA; 2 Department of Urology, Ottumwa Regional Health Center, Ottumwa, IA 52501, USA; dralameddine@hotmail.com

Our knowledge of voiding is derived from studies comprised of cisgender males and females, with relatively little known about the impact of sexual and gender minority status on voiding perceptions, symptoms, and function. Unfortunately, the increasing presence and diversity of sexual and gender minorities has not been met with a concomitant increase in urologic knowledge within this population. This study effectively captures the voiding experiences of this patient population, but does not discuss pathological and functional aspects that must be considered.

Incontinence should be characterized as stress urinary incontinence, urgency urinary incontinence, and mixed urinary incontinence, since different pathologies and treatment algorithms are associated with each. Risk factors for urgency incontinence include chronic cough, cystitis, dementia, depression, diabetes, drugs, endocrine disorders, neurologic disorders, mobility difficulty, and stool impaction [1]. The basic workup of incontinence begins with a history and physical exam, bladder diary, post void residual volume, and urinalysis [2]. Within the sexual and gender minority patient population, careful attention must be given to prior hormonal therapies, prior pelvic and abdominal surgeries, gender affirmation surgeries (GAS), reproductive organ status, a full neurologic exam, and a thorough pelvic/genital exam. In patients who have not undergone gender affirmation surgery, bladder training is considered the first line therapy for urinary incontinence. Bladder training includes timed voiding and overcoming the learned behavior of responding to the first sensation of urgency. If bladder training does not alleviate symptoms, then dietary modifications, weight loss, exercise, smoking cessation, pelvic floor muscle training, and biofeedback therapies may also help before pharmacological or surgical therapies are considered.

In patients who have undergone GAS, it is important to identify any signs/symptoms suggestive of urinary retention or obstruction. A complication after the creation of a neophallus and neourethra is urethral stricture, which can occur 25–58% in patients who underwent urethral lengthening [3]. Strictures can occur anywhere along the urethra, but occur most commonly between the phallic and fixed portions [4]. Patients can have urine accumulate proximally to strictures, which can be mistaken as incontinence. Some patients can manually massage this urine distally after voiding. Nevertheless, these complex cases should be evaluated by a urologist experienced with GAS and a high index of suspicion should be maintained for strictures and fistulae.

Despite the small sample size of 36 patients who described their lower urinary tract symptoms (LUTS) in this study, the largest proportion of patients that endorsed a prior urinary tract infection (UTI) was the queer group. Symptomatic UTI was not shown to be significantly related to sexuality, but was significantly related to AIDS status in one small study [5]. In this study, trans men between the ages of 18 and 25, but not 26 and 44, appeared to have the highest proportion of LUTS, suggesting that there may be some early structural or behavioral components to urinary urgency within sexual and gender minorities.

The focus group data underscore the influence of interactions and behaviors within restrooms on voiding distress. Simple improvements to the quality and privacy of the restroom infrastructure to change the environment may alleviate the psychological and functional complaints that these patients experience. This may ultimately reduce the amount of invasive urologic testing needed to evaluate these patients, which causes physical and emotional discomfort itself [6]. Nevertheless, adequate characterization and evaluation to identify functional abnormalities is still necessary and may warrant evaluation by a urologist.

## References

[1] Keilman L.J. (2005). Urinary incontinence: Basic evaluation and management in the primary care office. Prim. Care.

[2] DuBeau C.E., Kuchel G.A., Johnson T., Palmer M.H., Wagg A. (2010). Fourth International Consultation on Incontinence. Incontinence in the frail elderly: Report from the 4th International Consultation on Incontinence. Neurourol. Urodyn..

[3] Nikolavsky D., Yamaguchi Y., Levine J.P., Zhao L.C. (2017). Urologic Sequelae Following Phalloplasty in Transgendered Patients. Urol. Clin. North. Am..

[4] Lumen N., Monstrey S., Goessaert A.S., Oosterlinck W., Hoebeke P. (2011). Urethroplasty for strictures after phallic reconstruction: A single-institution experience. Eur. Urol..

[5] De Pinho A.M., Lopes G.S., Ramos-Filho C.F., Da Santos O., De Oliveira M.P., Halpern M., Gouvea C.A., Schechter M. (1994). Urinary tract infection in men with AIDS. Sex. Transm. Infect..

[6] Suskind A.M., Clemens J.Q., Kaufman S.R., Stoffel J.T., Oldendorf A., Malaeb B.S., Jandron T., Cameron A.P. (2015). Patient perceptions of physical and emotional discomfort related to urodynamic testing: A questionnaire-based study in men and women with and without neurologic conditions. Urology.

